# Rapid Changes in Transcription Profiles of the *Plasmodium yoelii yir* Multigene Family in Clonal Populations: Lack of Epigenetic Memory?

**DOI:** 10.1371/journal.pone.0004285

**Published:** 2009-01-28

**Authors:** Deirdre Cunningham, Jannik Fonager, William Jarra, Celine Carret, Peter Preiser, Jean Langhorne

**Affiliations:** 1 Division of Parasitology, National Institute for Medical Research, London, United Kingdom; 2 Pathogen Microarrays Group, The Wellcome Trust Sanger Institute, Cambridge, United Kingdom; 3 Bioscience Research Centre, Nanyang Technical University, Singapore; Federal University of São Paulo, Brazil

## Abstract

The *pir* multigene family, found in the genomes of *Plasmodium vivax*, *P. knowlesi* and the rodent malaria species, encode variant antigens that could be targets of the immune response. Individual parasites of the rodent malaria *Plasmodium yoelii*, selected by micromanipulation, transcribe only 1 to 3 different *pir (yir)* suggesting tight transcriptional control at the level of individual cells. Using microarray and quantitative RT-PCR, we show that despite this very restricted transcription in a single cell, many *yir* genes are transcribed throughout the intra-erythrocytic asexual cycle. The timing and level of transcription differs between genes, with some being more highly transcribed in ring and trophozoite stages, whereas others are more highly transcribed in schizonts. Infection of immunodeficient mice with single infected erythrocytes results in populations of parasites each with transcriptional profiles different from that of the parent parasite population and from each other. This drift away from the original ‘set’ of transcribed genes does not appear to follow a preset pattern and “epigenetic memory” of the *yir* transcribed in the parent parasite can be rapidly lost. Thus, regulation of *pir* gene transcription may be different from that of the well-characterised multigene family, *var*, of *Plasmodium falciparum*.

## Introduction

*Plasmodium* infections can result in chronic infections in most vertebrate hosts, and parasites may persist for months or years. In spite of a vigorous host immune response, sterile immunity is rarely achieved. Variable surface antigens (VSA) expressed at the surface of the parasitised red blood cell (pRBC) help the parasite evade host immunity [1]–[4] and may explain how these chronic infections are maintained. Multigene families coding for VSAs exist in several malaria parasite species [5], and paralogs and orthologs are found within and across these species [6]–[8]. Waves of parasitaemia have been described in *P. falciparum* infections in humans, reflecting the switching of expression of such parasite antigens [9]. In *P. falciparum*, several families of variant antigens have been identified (including *vars*, *rifins*, *stevors*), although only the understanding of the *var* gene family is well advanced (reviewed by [10]. There are no known strict homologues of the *var* family in other *Plasmodium* species infecting humans, or rodents, and while there is some evidence that the *SICAvar* gene family of the simian parasite *P. knowlesi* may have some evolutionary relationship to *var* [11] this is not an easily accessible system to study the complex interaction between variant antigens and the host immune response.

*Plasmodium vivax* also establishes long lasting chronic infections, and its genome contains a large multigene family, *pir* (Plasmodium interspersed repeat), which could play a role similar to *P. falciparum var*, and be involved in immune evasion. In *P. vivax* there are approximately 350 *pir (vir)* in the genome [12], and homologues have been identified in *Plasmodium* infecting rodents and monkeys; *P. berghei (bir)*, *P. chabaudi (cir)*, *P. yoelii (yir)*, *P. knowlesi (kir)* [6], [7], [13]–[15]. Consistent with a role in antigenic variation and immune evasion, PIR proteins have been demonstrated on, or close to the surface membrane of erythrocytes infected with *P. vivax*, *P. chabaudi*, *P. yoelii and P.berghei* [14], [16]–[18], and switching of *yir* transcription has been observed in response to a host immune response [17].

In *P. vivax* and *P. yoelii*, 5 to 6 distinct subgroups of *pir* genes or their deduced proteins have been identified, and many *pir* genes from the different subgroups appear to be expressed in individual infections [17], [19]–[22]. There is some restriction or control on *pir* gene expression, however, as non-overlapping sets of PIR proteins or transcripts can be detected at different life cycle stages of *P. berghei* and *P. vivax* [23], [24]. As yet it is not clear whether the many *pir* genes transcribed are the result of many individual parasites transcribing one or a few *pir* genes or due to each parasite transcribing many or all *pir*, and whether this non-overlapping expression is absolute, or can change from infection to infection.

In *P. falciparum*, transcription of *var* genes is tightly regulated in a stage-specific manner, with multiple *var* being transcribed in ring stage parasites, whereas only a single member of *var* is transcribed in a single trophozoite infected cell [25], [26]. Similarly, only 1–2 *stevor* transcripts can be detected in single micromanipulated parasites [27]. Furthermore, transcription of both *var* and *stevor* is stably maintained by an epigenetic memory in clonal parasite populations [28], [29] with only low levels of transcript switching being observed from one generation to the next.

Here, we have investigated *yir* transcription both in single micromanipulated parasites as well as in parasite populations expanded from a single injected parasite. We show that, similar to *vir* in *P. vivax* [20] and *var* and *stevor* in *P. falciparum*, only one to three *yir* transcripts can be detected in single parasitized erythrocytes. In contrast, microarray analysis of clonally expanded parasites shows transcription of a large number of different *yir*, consistent with rapid switching or a lack of epigenetic memory. This data provides clear evidence that regulation of expression of *yir* genes is very different to that observed for the *var* or *stevor* gene family in *P. falciparum* maintained *in vitro*, indicating that antigenic variation mediated by *yir* does not involve the sequential activation of one gene after another but the exposure of many YIR variants to the immune system at the same time. Crucially, the regulation of *yir* is very consistent with the current available data for *vir* gene regulation in *P. vivax*, making this a suitable model system to investigate the broader biological role of this multigene family. This study seeks to characterise the repertoire of *yir* transcripts seen in single cells and to contrast these with that seen in the progeny of cloned parasites replicating in immunodeficient mice.

## Results

### Many *yir* genes are transcribed in an erythrocytic stage infection with *P. yoelii*

**Figure 1:**
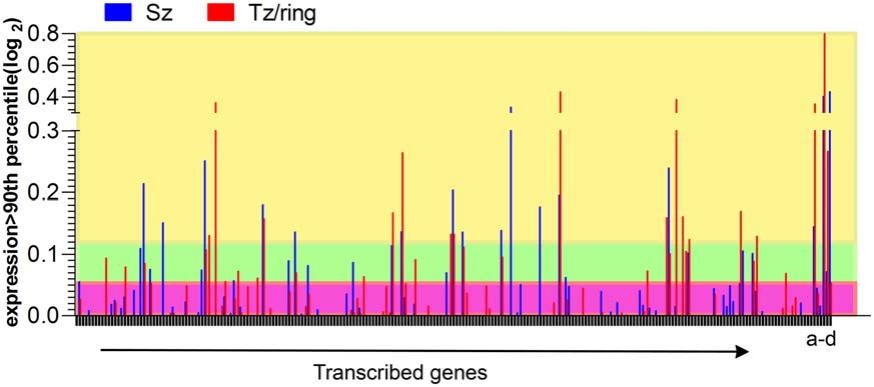
Microarray gene expression data from schizont and ring /uninucleate trophozoite stage parasites from a primary *P. yoelii* infection. *Yir* gene expression signals (log2) in schizont (blue) and ring /uninucleate trophozoite (red) stage parasites for each gene are paired and shown as bars, for the complete set of *yir* genes analyzed in this experiment. Positive gene expression values, above the 90^th^ percentile cut-off in the starter population, are shown. Data points for all *yir* (226) genes displaying positive gene expression values, in the samples analysed, are included in the graph (see also Supplementary Table S1 for gene identity (ID)). Cut-off levels for genes transcribed at high (yellow), medium (green) and low (purple) levels are indicated by shading. Expression of several non-*yir* genes (*Pc*fam homologues (a), multiple banded antigen (b), ribosomal subunit protein L23 (c), β-tubulin (d)) is shown for comparison.

The Affymetrix PFSANGER microarray contains, in addition to the *P. falciparum* genome, specific probes for members of the *pir* family including *P. knowlesi*, *P. vivax*, *P. yoelii*, *P. chabaudi*, and *P. berghei* species. To investigate the specific transcription of the *yir* genes of *P. yoelii*, different life cycle stages of intra-erythrocytic parasites obtained from primary (starter) infections of RAG2−/− BALB/c mice were considered. As a first step, the PFSANGER *yir* gene set was validated by hybridizing *P. yoelii* 17× genomic DNA, establishing 578 *yir* genes as the working set for which mRNA expression will be evaluated in different conditions. Analysis of RNA derived from all intra-erythrocytic parasite samples showed low level transcription of 226 *yir* genes (figure 1, 113 *yir* transcribed in starter parasites are shown; Supplementary Table S1 includes microarray data for these samples, colour coded according to transcription level). Three hundred and fifty-two genes were below the cut off level in all samples. Interestingly the *yir* genes expressed in ring/trophozoite or schizont infected erythrocytes were not necessarily the same. Transcription of some non-*yir* housekeeping genes were expressed at substantially higher levels than *yir* e.g. *Pc*fam homologue, multiple banded antigen, ribosomal subunit protein L23, and β–tubulin, possibly reflecting the fact that only subsets of parasites transcribe a particular *yir* whereas housekeeping genes are transcribed by every parasite. Although the microarray may not have been sensitive enough to detect very low transcription levels, this global analysis of *yir* transcription supports previous reports that many, but not all, *yir* genes are transcribed in the course of an infection [17], [22], (Preiser, unpublished data).

**Figure 2:**
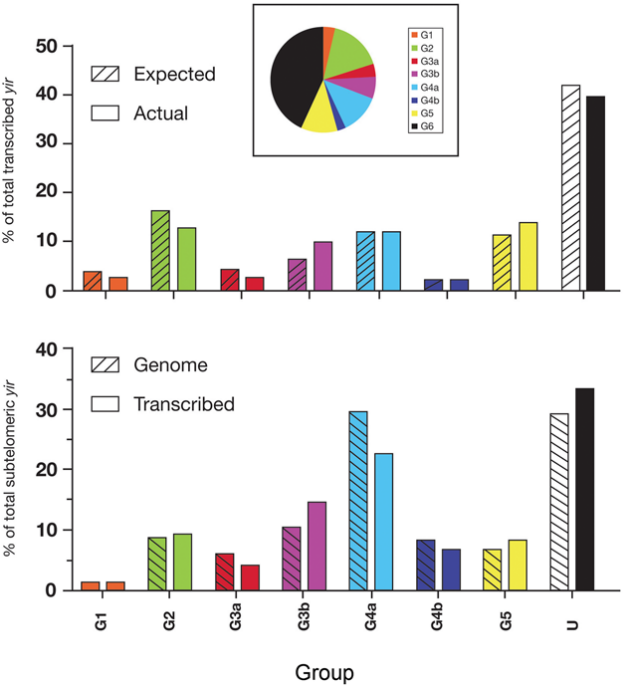
Group assignment and chromosomal location of *yir* genes transcribed in blood stage infection of *P. yoelii*. The proportion of transcribed *yir* genes (a) and transcribed subtelomeric *yir* genes (b) in each group is compared to the proportion expected from their representation in the *P. yoelii* genome. Inset: Proportion of *yir* genes of each group in the genome (adapted from Fonager *et al*, 2007).

*Yir* genes have been found in telomeric as well as subtelomeric locations (i.e. contigs assigned to a telomeric or a subtelomeric location by TIGR), and approximately 56% of them can be classified into five well-supported groups, with the remaining 44% being too divergent to be grouped (unassigned) [22]. It had been speculated that the groupings and chromosomal location could reflect families of *pir* genes with different functions and differential transcription in the various life cycle stages [21], [22]. However, in this microarray analysis we found no preferential transcription of *yirs* (within the top 10^th^ percentile), compared with the group distribution of all the genes (figure 2a). We also investigated whether any of those expressed *yir* genes were preferentially distributed at telomeric or subtelomeric locations but we failed to find any enrichment (figure 2b). Thus, it appears that, by and large, the *yir* genes are randomly expressed across the genome in intra-erythrocytic parasite life cycle stages, regardless of their chromosomal localization and sequence features.

### Individual intra-erythrocytic parasites transcribe only one to three *yir* genes

**Figure 3:**
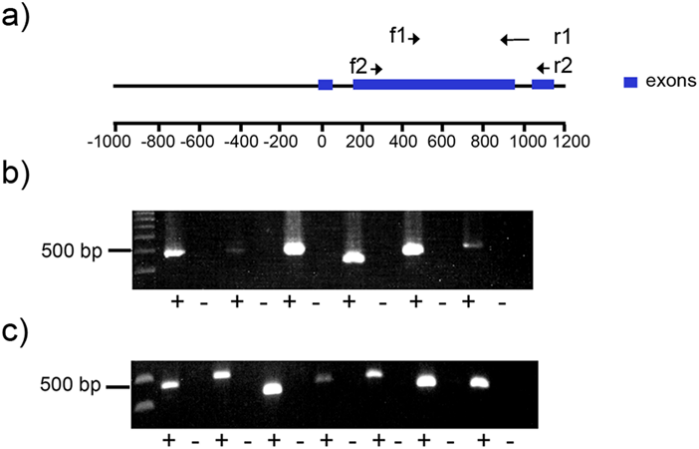
RT-PCR on single micromanipulated schizonts and uninucleate trophozoites. a) Location of the conserved *yir* primer pairs in a genomic setting. f1 (forward primer 1)/r1 (reverse primer 1) and f2/r2 pairs are shown with black arrows, while the *yir* gene structure is shown in blue, sizes are indicated below the gene in bp. b) Single cell RT-PCR on six micromanipulated schizonts (+ lanes 2–12). c) Single cell RT-PCR on seven micromanipulated uninucleate trophozoites (+ lanes 2–14). Expected product sizes were 500–600 bp, controls were reactions from which reverse transcriptase enzyme was omitted (− lanes 3–13 for schizonts and 3–15 for trophozoites). Marker was 100 bp DNA ladder.

The microarray analysis performed extends our previous work using RT-PCR which showed that a large number of different *yir* were transcribed at the same time during an infection [17]. To establish whether this is due to single parasites transcribing many *yir* or alternatively due to different parasites each transcribing a different but restricted set of *yir*, single erythrocytes infected with schizonts or trophozoites were selected by micromanipulation, and cDNA synthesised from amplified RNA was analysed by RT-PCR with a broad-range conserved primer set. Figure 3 shows the amplified products obtained from each of six schizonts and seven trophozoites. Parasites within individual infected RBC transcribed only 1 to 3 different *yir* genes at a given time (Table 1). This was in stark contrast to the large repertoire of transcribed *yir* genes in the parasite population as a whole, suggesting that transcription of this multigene family was indeed tightly controlled in individual cells.

**Table 1:** *Yir* transcripts found in individual micromanipulated erythrocytes infected with *P. yoelii* at the schizont or ring/trophozoite stage of development.

Cell^1^	Transcript(s)^2^			yir group^3^		
Sz1	PY04580^4^			u		
Sz2	PY05822	PY06340^4^		u	G5	
Sz3	PY02523	PY03973,	PY04580^4^	u	u	u
Sz4	PY02688			u		
Sz5	PY05828	PY00351		G1	u	
Sz6	PY05828^5^			G1		
Tz1	PY00351			u		
Tz2	PY02298			u		
Tz3	PY02688			u		
Tz4	PY07466	PY03966		G2	u	
Tz5	PY06939	PY03193	PY02263	u	u	u
Tz6	PY02688			u		
Tz7	PY02688			u		

1 Infected erythrocyte containing schizonts with 5 or 6 nuclei (Sz) or uninucleate trophozoite (Tz).

2 Detected using conserved splice-site spanning primer set f1/r1 (described in Supplementary Table S3).

3 As determined from [22] and shown in figure 2, Groups (G) 1 to 5 and unassigned yir genes (u).

4 Subtelomerically located.

5 Unspliced (as determined by sequencing).

In total, fourteen transcribed genes were identified by sequencing (Table 1), one *yir* only from each of the groups G1, G2, G5, with the remaining 11 being unassigned. Splicing was confirmed for several genes (5 transcripts within 4 schizont infected cells (results not shown)). As expected from the microarray data, location was again unimportant for transcription in individual cells, with only three transcribed genes (∼20%) being found at the subtelomeres.

### Rapid switching of *yir* genes during an infection

**Figure 4:**
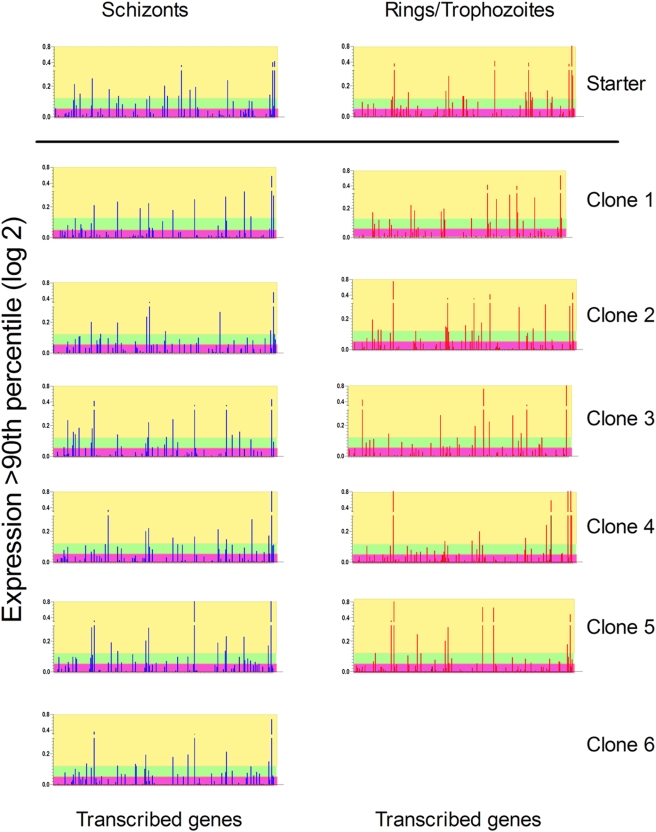
Microarray analysis showing changes in *yir* transcription patterns in cloned parasites. a) Transcription pattern of *yir* genes in the starter population of *P. yoelii* ring/uninucleate trophozoite stages (R, in red) and schizont stage (S, in blue) parasites. Each gene is indicated as a bar, expression signal is as defined in figure 1; b) Comparison of the transcription profile of 578 *yir* genes used as the repertoire of transcribed *yir* genes derived from infections initiated with a single infected erythrocyte (by limiting dilution). The repertoire of transcribed *yir* genes in schizont and ring/uninucleate trophozoite stage parasites was determined after ∼10 days of infection. The microarray fingerprints represent the repertoire of genes transcribed from individual mice infected with a single infected erythrocyte obtained from the initial infection (top array and figure 1). [See also Supplementary Table S1, part b for transcribed genes common to all samples].

Hierarchical transcription of genes has been observed for members of other multigene families of *Plasmodium* [26], . Furthermore, switching rates are relatively low and cloned parasites predominantly express a single member of a multigene family for many rounds of replication [28], [31]. To investigate whether this was the case for *yir* genes, RAG2−/− mice were infected with a single *P. yoelii*-infected RBC, and the transcription patterns of *yir* genes in the parasite progeny of these “clones” analysed by microarray (figure 4 and Supplementary Table S1). The use of immunodeficient mice allows us to address the question of whether an infection arising from a single infected cell will transcribe the same family members, in the absence of any selection pressure applied by the developing specific immune responses. None of the *P. yoelii* infections initiated by a single infected erythrocyte gave rise to transcription of a single or few predominant *yir* genes; rather, in all cases, a significant number of genes were transcribed regardless of whether parasites were analysed at the ring/uninucleate trophozoite stage or at the schizont stage, and overall transcription levels were comparable with those of the starter population (figure 1). However, in these clonal infections the transcription patterns of *yir* after approximately 10 to 12 replication cycles were not identical with that of the initial starting population from which they were cloned. Overall, only 8 *yir* (of the 226 detectable by microarray) were expressed at all stages in the 6 clones analyzed, as well as the starter population, albeit with different levels of transcription. In addition, in the starter population and in the clones, the *yir* transcription profile differed between immature and mature stage parasites, with the same *yir* gene seldom being expressed at the same level in the same clone and at both time points, indicating that different sets of *yir* are transcribed during these stages. Comparison of the set of *yir* detected in either ring/trophozoite or schizont stages showed no association of any *yir* transcript with a particular stage but revealed that the expression of a given gene set occurs randomly, rather than in a hierarchical or pre-programmed manner (figure 4 and Supplementary Table S1). Indeed, for identical genes when expression of a particular *yir* was high in ring/trophozoite and low in schizont in one clone, this pattern was inverted in a different clone. As we know little of the stability of *yir* RNA at present the data presented refers to transcripts detected within the developmental stages analysed.

Six *yir* genes (PY05826, PY03177, PY04021, PY01996, PY02298, PY03045), detected at different levels, or undetected (PY03045), by microarray in the starter population, in ring/trophozoite- or in schizont-stage parasites of the clones, were selected for more detailed analysis by qRT-PCR. Gene-specific primers for these selected genes resulted in comparable amplification efficiency as shown by amplification of genomic DNA (data not shown). qRT-PCR on cRNA from trophozoite/ring-stage and schizont-stage infected RBC confirmed that transcription of the individual *yir* genes between the different parasite stages and between clones was variable. Overall, transcription levels of *yir* were approximately 2 logs lower than that of the housekeeping gene, β–tubulin, or of merozoite surface protein-1 (MSP-1), in general agreement with the results of the microarray data (not shown).

**Figure 5:**
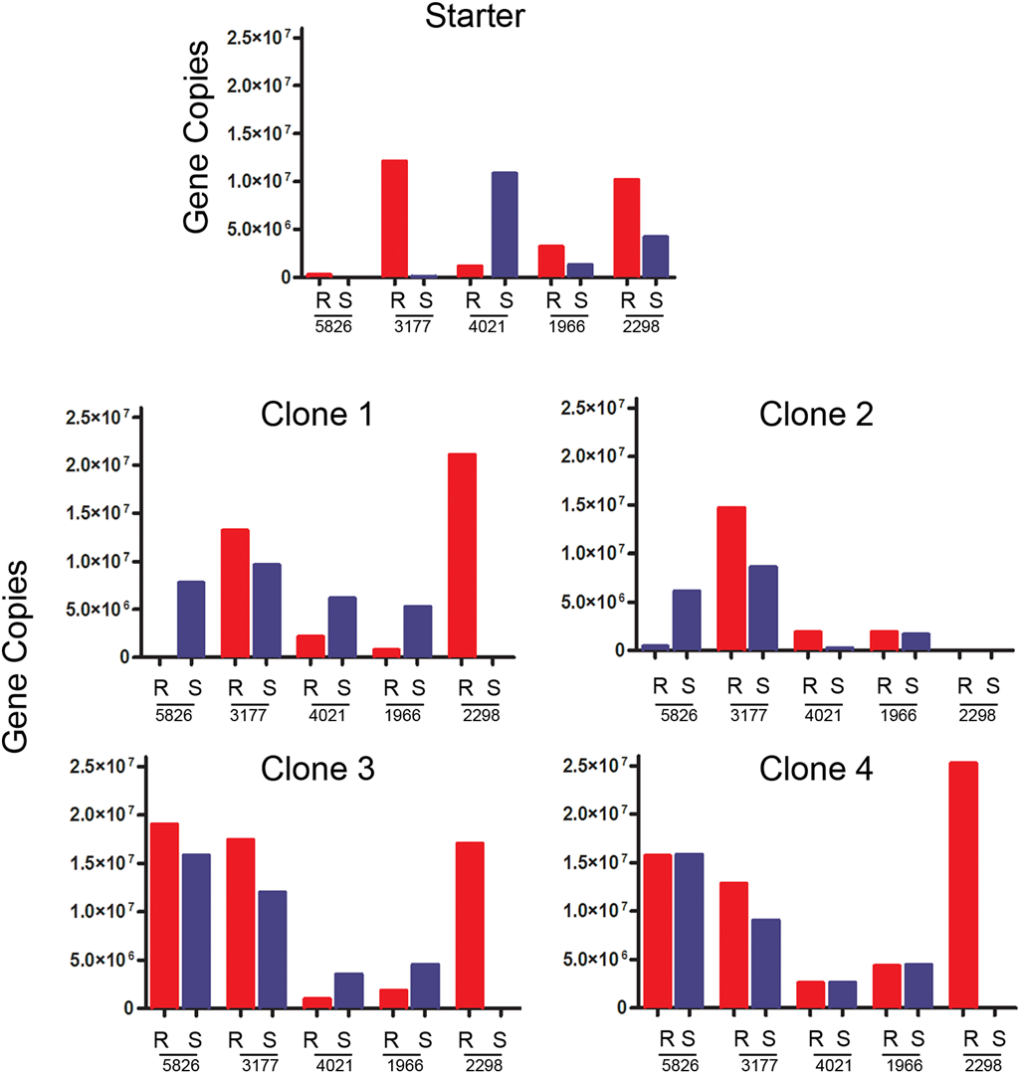
Quantitation of selected genes by qRT-PCR in stage-separated parasites. Comparison of transcription levels of the *yir* genes PY05826, PY03177, PY04021, PY01966 and PY02298 in stage separated parasites (Rings and uninucleate trophozoites (R) are in red and Schizonts (S) are in blue) isolated from four parasite clones (Clones 1–4), expressed as number of copies transcribed /1×10^−9^ g β-tubulin.

Most strikingly, analysis of the RNA of the progeny of 4 individual clones showed that, as in the microarray, each clone exhibited a different profile for 5 of the *yir* genes, (PY03045 was not detectable in any of the clones). Furthermore, expression of each *yir* showed no consistent pattern between schizont and ring/trophozoite stages (figure 5). For example, PY05826 transcripts were present only at a very low level in ring/trophozoite stage parasites in the starter population and not detectable in schizonts. However, in two of the clones (Clones 3 and 4) the same *yir* was expressed in both rings/trophozoites and schizonts but in the 2 other clones (Clones 1 and 2) it was preferentially detected in the schizont preparation (figure 5). PY03177 was only detectable at ring/trophozoite stage in the starter population, but was expressed with a similar pattern in both stages in all clonal populations.

Both qRT-PCR and microarray data taken together show that i) many *yir* genes are transcribed in an infection, ii) there is a rapid switch of expression for the expressed *yir* and iii) no obvious pattern of expression was observed even when the infection was initiated with a single parasite. The low levels of transcription of individual *yir* in the population are probably due to the fact that many *yir* are transcribed, but each parasite in the population transcribes only a very restricted number of *yir*.

## Discussion

Here, we have investigated changes in the transcriptional profile of the *pir (yir)* multigene family *in vivo* during a *P. yoelii* infection of mice. We show that although individual intra-erythrocytic parasites only transcribe 1 to 3 genes at a time, these “founder” transcripts are rapidly lost in infections initiated with a single infected erythrocyte. Instead, a wide range of different *yir* transcripts can be detected in the infection after only 10–12 generations. There is no apparent order in which transcripts are being made as 5 different clonal infections show a completely different transcription pattern with only a minimal overlap in the genes transcribed at high levels. Our observation that individual parasites in a population transcribe different *yir* and therefore would be expected to express different YIR contrasts with recent work in *P. knowlesi* that suggested that post-transcriptional silencing of variant antigen transcript ensures that only a small set of *SICAvar* are expressed in a population despite high levels of transcripts being detected for other *SICAvar* [32].

Our current understanding of the regulation of expression of *Plasmodium* multigene families is based mainly on studies performed in *P. falciparum*. Mutually exclusive transcription of both *var* and *stevor* has been observed in cultured *P. falciparum* laboratory strains [26], [29]. However, in cultures, once an infected RBC has been selected for surface expression of a single *var*, expression is relatively stable in the absence of selection, and the dominant *var* transcripts can be detected over many generations, while there is a gradual expansion of parasite subpopulations expressing other *var* [28], [33]. The strongest evidence for mutually exclusive *var* expression comes from the investigation of the dynamics of transcriptional switching using episomally transfected parasites [33], [34]. These studies have shown that *P. falciparum var* gene activation within a clonal parasite population does not follow a predetermined order, but instead the transcriptional switching rate depends on the on/off rate of the genes and chromosomal location, with subtelomerically located genes exhibiting the highest switching rate, regardless of switching history [33], [31]. Variation in gene expression between clones has also been demonstrated for the *stevor* and *Pfmc-2TM* families, which also exhibit variation in switching rates between isogenic clones [29].

Our analysis of *yir* transcriptional changes *in vivo* described here and previously [17] suggests that regulation of *pir* transcription is different from the *var* regulation observed in cultured parasites. Indeed, the pattern observed for *yir* reflects observations made in samples obtained from either patients infected with *P. vivax* or mice infected with *P. berghei* where many *vir* and *bir* transcripts are detected [20], [23]. Similar to our findings, analysis of *var* transcripts in parasites obtained directly from *P. falciparum* infected patients also shows clearly that many members of both *var* and *stevor* are being transcribed [35], [36], in apparent contrast to the observations of cultured *P. falciparum* [25], [26]. Indeed, a human *in-vivo* volunteer study showed transcription of all *var* genes in cultures derived from first generation parasites whereas cultures from second and third generation parasites transcribed a smaller subset of *var* genes [37]. These data could indicate that switching of *var* genes occurs at higher rates in natural infections than in cultured parasites, that the repertoire is quickly dominated by the expansion of more rapidly growing parasites expressing a more limited repertoire or that the *var* and *stevor* transcript repertoire detected in patients is derived from multiple infections running concurrently. In our studies, the many *yirs* transcribed *in vivo* are not the result of multiple infections as even infections initiated from a single parasite transcribing 1 to 3 *yirs* give rise in a short time to transcription of many *yir* in an immuno-compromised mouse.

Work both on *yir* genes [22] as well as *vir* [21], [12] have shown that *pir* genes can be grouped into related subgroups, suggesting distinct functions of *pir* during the life cycle of the parasite. The finding that subsets of PIR proteins of another rodent malaria parasite, *P. berghei*, have been detected exclusively at particular life cycle stages [23], and that BIR proteins are associated with both the erythrocytic membrane and membrane microdomains, or lipid rafts [18] would support this view, although no functions have yet been elucidated in these cases. Our analysis of the complete repertoire of *yir* genes transcribed during the erythrocytic stages of *P. yoelii*, however, did not reveal preferential transcription of particular subsets of *yir* genes, for the most part, as defined either by phylogenetic group or by chromosomal location [22] and this agrees with the observations made for *vir* transcription in *P. vivax* [24] showing that although non-overlapping sets of *vir* are transcribed in early (ring) and late (schizonts) stage parasites, there is no evidence that distinct phylogenetic groups of *vir* are linked to stage specific expression. In fact, in different patient samples the transcription pattern of distinct *vir* can switch from ring to schizont stage or *vice versa* [24].

We previously observed alternative splicing occurring in a phylogenetic subset of *yir* transcripts [22], and some of these splice-variants could clearly block YIR protein translation. Although we show here that no particular group of *yir* genes is predominately transcribed in the asexual blood stages, alternative splicing might lead to distinct PIR expression profiles observed in different stages [23]. Clearly, a more extensive analysis of *yir* expression in the other stages of the *P. yoelii* life cycle and location of the YIR proteins within different parasite stages is needed to address whether functionally distinct subsets of YIR exist.

Despite micromanipulation and PCR on single infected RBC we cannot determine whether *yir* transcription is mutually exclusive in individual cells or whether transcription more resembles that of the *rifin* genes, where subtypes A and B can be co-expressed in a single cell [38]. There was clearly transcription of a very limited number of *yir* genes by individual *P. yoelii* infected RBC. Although we cannot rule out the possibility that more *yirs* could be expressed, as the microarray coverage for the *pir* genes was not comprehensive, the expression profile agrees with earlier observations on *vir* gene expression of *P. vivax*, where *vir* gene transcripts from two subfamilies of *vir* were detectable in a minority of single infected RBC [20].

Epigenetic mechanisms are thought to be the main factors that regulate mutually exclusive transcription of *var* and *stevor* in *P. falciparum* and ensure the inheritance of transcription patterns from one generation to the next [39], [40]. The data presented here showing transcription of only 1 to 3 *yir* genes in a single parasite suggest that an epigenetic silencing mechanism is operating. However, unlike *var* transcription in cultured *P. falciparum* such an epigenetic imprint appears to be rapidly lost from one generation to the next. Currently, it is not known whether *P. falciparum* in natural infections retains the strong epigenetic imprint observed in cultured parasites or shows an equally rapid loss of memory as observed here for *yir*.

Our previous finding that some YIR proteins can be found on the surface of the infected cell, and that the presence of an intact immune system is important in the modulation of the transcribed repertoire [17] suggests a role for some members of this family in antigenic variation on the surface of the infected RBC. The pattern of *yir* transcription observed here in clonal infections is not compatible with current thoughts on antigenic variation in pathogens where waves of dominant transcripts would be expected during the course of an infection. Instead it appears that the parasite expresses a large repertoire of different *yir* simultaneously, albeit on different infected cells. Even if only a proportion of these genes give rise to functional proteins, the host immune system would be challenged by a large number of highly related antigens and thus it is possible that no antigen is present at a level sufficient to induce a rapid immune response. In addition, the establishment of a unique YIR profile at each infection would also ensure parasite survival in multiple infected hosts. This allows YIR to act as a “smoke screen” slowing down the acquisition of protective immunity in the host, ensuring maintenance of the infection but with some parasite control, advantageous to both host and parasite. All *yir* may not be expressed at the same level, and if there are more parasites expressing a particular type of YIR compared with others, immunity may develop first against the dominant YIR expressed, while those YIR genes expressed at lower levels are not recognized. Under these circumstances one would expect that the transcribed *yir* repertoire becomes more and more restricted as the infection progresses, possibly leading to only a single transcript being detected in the later stages of a chronic infection. Such a mechanism could readily be investigated in *P. chabaudi* infections of mice, or in rodent malaria infections in their natural host, *Thamnomys rutilans*, where infections are chronic for several months. The high similarity in *pir* transcription for both *P. yoelii* and *P. vivax* would strongly support a conserved functional role for this gene family both in rodent and the human parasites. Clearly, an analysis of YIR protein expression within or on the parasite or parasite-infected cell and in different stages of parasite development is the necessary next step to evaluate the function of the *pir* multigene family in *Plasmodium* infections and will give important new insights into the biological role of this gene family in *P. vivax*.

## Materials and Methods

### Mice and Parasites

Male and female BALB/c mice, and BALB/c mice with a targeted disruption of the RAG2 gene (RAG2−/−) [41] were bred and maintained in filter racks on sterile bedding, food and water at the National Institute for Medical Research (NIMR).

*P. yoelii* 17× A parasites were derived from a cloned line (A) provided by Dr D. Walliker, as described previously [42]. Parasitaemia was monitored by daily microscopic analysis of methanol-fixed Giemsa-stained thin blood smears. BALB/c and RAG2−/− mice were infected intraperitoneally (i.p.) with *P. yoelii*. For fractionation of parasites by stage for micromanipulation, parasite preparations containing predominantly *P. yoelii* rings, trophozoites and schizonts were obtained from a discontinuous Nycoprep (Nycomed Oslo, Norway) step gradient composed of equal volumes of a 27.6% Nycodenz working solution in TrisHCl pH 7.4 diluted to 55 and 80% in RPMI 1640 medium. For analysis of the parasite populations from mice infected with a single parasite, cell populations enriched for mature parasite stages (mature trophozoites and schizonts) were separated from immature trophozoites/rings on 55% Nycoprep cushions. The relative numbers of cells, by stage, in each band was determined from Giemsa stained thin blood films. With one exception (clone 5) separation of parasite stages on the 55% Nycoprep cushion resulted in an enrichment of schizonts (52.5–78.7%) in the (top) layer excluded from the Nycoprep. However, this cell population also consisted of 18–42.5% mature trophozoites. Rings and immature trophozoites were virtually absent (0.2–5.8%). Parasitized erythrocytes which formed the pellets in the cushions were enriched for uninucleate parasites (rings/immature trophozoites/mature trophozoites - 86–98%) but contained very low numbers of schizonts (1.3–14%) [see Supplementary Table S2 for exact proportions of each developmental stage in stage separated parasites).] All infections were initiated from the same founder or ‘starter’ parasite stabilate. Parasites were cloned by limiting dilution into RAG2−/− mice and the infection monitored by examination of Giemsa-stained thin blood films. Clones were prepared by limiting dilution rather than micromanipulation for logistical reasons; the former technique being favoured when larger numbers of clones are desired. The infections were initiated from dilutions containing (theoretically) 1.0 or 0.5 parasites. The number of successful infections over the total number of inoculations was within the numbers predicted by the zero order term of Poisson distribution and this, along with our own observations on the parasite material used, indicate that the majority of the clones would have derived from an erythrocyte infected with a single uni-nucleate parasite [43]. Parasites were collected after 8–10 days for preparation of parasite RNA.

### Micromanipulation of single-cell parasites and single cell RT-PCR

Schizont- and trophozoite-enriched *P.yoelii*-infected red blood cells were obtained as described above, pelleted cells (500×*g* for 10 min at room temperature) were washed twice in Krebs buffered saline, and single parasites were micro-manipulated as described previously [44].

Single cells were lysed and RNA released by heating parasites at 93°C for 3 minutes [45]. cDNA was prepared from single cells by direct addition of the crude lysate to the Super SMART™ PCR cDNA amplification kit (Clontech, BD) followed by first-strand cDNA synthesis and aRNA amplification for 40 cycles, performed according to the manufacturers instructions. Following cDNA synthesis, *yir* specific primers were used to amplify *yir* genes by PCR. Primer sequences (conserved sets) and amplification conditions are detailed in Supplementary Table S3. Conserved primer sets designed to amplify *yir* genes across the phylogenetic tree and validated on genomic DNA were initially used (data not shown) in order to avoid any primer bias but the conserved set f1/r1 were used subsequently as the results obtained were identical. Cloning and sequencing was performed as described previously [22] with at least ten clones being sequenced per amplification.

### RNA extraction and Quantitative Real Time PCR

Total RNA was extracted as described by Kyes *et al.*, 2000 [46]. Prior to quantitative real-time PCR, RNA samples were digested with TURBO DNase (Ambion Inc), according to the manufacturer's instructions, followed by cDNA synthesis from 1 µg RNA, primed with random hexamers (Clontech) using superscript™ II reverse transcriptase (Invitrogen) according to the manufacturer's instructions. PCR reactions were prepared in Absolute™ SYBR® Green mix (containing Thermo-Start™ DNA polymerase and ROX Dye)(Abgene), containing 0.2 µM each primer and 1 µl cDNA (of a 40 µl reaction), and amplification performed on an ABI Prism® 7000 Sequence Detection System (Applied Biosystems). Cycle conditions were 50°C, 2 min; 95°C, 15 min; 35 cycles of 95°C, 15 s; 60°C, 1 min. Gene specific primers are listed in Supplementary Table S3.

Primers were validated on genomic DNA to ensure amplification efficiencies were comparable and plasmid standards were constructed for each gene and included in each assay.

### Microarrays

PFSANGER Affymetrix arrays are high-density 8-µm custom 25-mer oligonucleotide arrays, whose tiling-like design was based on the *P. falciparum* genomic sequence released in January 2005 (www.genedb.org), as described previously [47]. Of interest and in addition to *P. falciparum* probes, the PFSANGER array contains 45478 oligonucleotides that have been designed against 815 different *P. yoelii yir* genes. *P. yoelii* total RNA was reverse transcribed and biotin-labelled as cRNA, using the GeneChip IVT Labelling kit, as recommended by Affymetrix (www.affymetrix.com). Samples were hybridised, washed, stained and scanned as described previously [47]. Prior to transfer the files into R/Bioconductor [48] for analysis, we modified the original CDF for our purpose, focusing on the additional *pir* genes, using makeCDFenv package (Rafael A. Irizarry, Laurent Gautier, Wolfgang Huber and Ben Bolstad (2006). makecdfenv: CDF Environment Maker. R package version 1.18.0). Modified CDF environment and raw CEL files can be retrieved from ArrayExpress under the accession number E-TABM-537. The raw CEL files were then normalised using RMA [49]. A 90^th^ percentile cut-off was applied, calling a gene expressed if at least 5 probes were mapped to it and its median summarized signal value was over the threshold. To ensure that the *yirs* not detected as expressed were still present on the PFSANGER array, we performed a genomic DNA hybridization using *P. yoelii* 17× A used in this experiment, as previously described [50].

## Supporting Information

Table S1 Microarray data for 226 yir genes, colour coded according to transcription level. a) Transcribed genes All yir genes showing positive gene expression values above the 90th percentile cut off, with corresponding data values for each sample, are listed. Data points for genes with low (90th–94th percentile; medium (95th–97th percentile) and high (>98th percentile) transcription levels are colour coded as indicated. b) High and Medium transcription in all clones All yir genes exhibiting high/medium gene expression in all clones are listed, with colour coding as indicated
(0.08 MB XLS)

Table S2 Proportion of parasites at each developmental stage obtained from parasites fractionated over a Nycodenz cushion
(0.08 MB DOC)

Table S3 Gene specific primer pairs for Q-RTPCR and single cell RTPCR
(0.03 MB DOC)
