# Supplementary material for: Rapid Changes in Transcription Profiles of the Plasmodium yoelii yir Multigene Family in Clonal Populations: Lack of Epigenetic Memory?
Source: PLoS One. 2009 Jan 28;4(1):e4285. doi: 10.1371/journal.pone.0004285 (PMC2628738; doi:10.1371/journal.pone.0004285)
Supplement: Table S2 — Proportion of parasites at each developmental stage obtained from parasites fractionated over a Nycodenz cushion (0.08 MB DOC) [file pone.0004285.s002.doc]

**Supplementary Table 2** Proportion of parasites at each developmental stage obtained from parasites fractionated over a Nycodenz cushion.

| **Sample** | **Fraction** | **Percentage** | |
| --- | --- | --- | --- |
|  |  | **Rings and Uninucleate trophozoites^1^** | **Schizont^2^** |
| Starter | T-layer | 29.8 | 70.2 |
|  | Pellet | 98.5 | 1.5 |
|  |  |  |  |
| Clone 1 | T-layer | 21.3 | 78.7 |
|  | Pellet | 97.7 | 2.3 |
|  |  |  |  |
| Clone 2 | T-layer | 42.7 | 57.3 |
|  | Pellet | 95.2 | 4.8 |
|  |  |  |  |
| Clone 3 | T-layer | 31 | 69 |
|  | Pellet | 86 | 14 |
|  |  |  |  |
| Clone 4 | T-layer | 40 | 60 |
|  | Pellet | 94 | 6 |
|  |  |  |  |
| Clone 5 | T-layer | 47.5 | 52.5 |
|  | Pellet | 95.5 | 4.5 |
|  |  |  |  |
| Clone 6 | T-layer | 40 | 60 |
|  | Pellet | 98.7 | 1.3 |

Parasite stages fractionate into rings and immature trophozoites^1^ (uninucleate) and mid-stage/mature (uni-nucleate) trophozoites^1^ and schizonts^3^ (>1 nuclei) as shown:

**
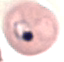

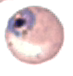

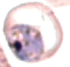

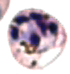

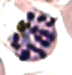
**

1) Rings/uninucleate Tz 2) Schizont

**To initiate single cell infections:-**  blood used was taken at a relatively early stage of infection in a RAG2-/- BALB/c mouse when the incidence of erythrocytes containing more than one parasite was expected to be low i.e. approximately 2.7%. This parasite population comprised 91% uni-nucleate parasites (72% Rings, 19% trophozoites) thus 9% multi-nucleate (> 1 nuclei) schizonts. Data were obtained from counts of 7 replicate slides.
