# Supplementary material for: Rapid Changes in Transcription Profiles of the Plasmodium yoelii yir Multigene Family in Clonal Populations: Lack of Epigenetic Memory?
Source: PLoS One. 2009 Jan 28;4(1):e4285. doi: 10.1371/journal.pone.0004285 (PMC2628738; doi:10.1371/journal.pone.0004285)
Supplement: Table S3 — Gene specific primer pairs for Q-RTPCR and single cell RTPCR (0.03 MB DOC) [file pone.0004285.s003.doc]

#### Supplementary Table 3 Gene specific primer pairs for qRT-PCR and single cell RT-PCR

| **Gene** | **Sequence** | **Location** | **Amplification conditions** |
| --- | --- | --- | --- |
| Beta tubulin f Beta tubulin r | AGCAGGCCAATGTGGTAATC ACCTGCACGAACACTATCCA | PY05711 24-44 PY05711 217-237 | 60°C annealing/extension; 35 cycles |
| PY03177f PY03177 r | GAACTGATTTGCTGAATATG GGTCATTGTTAAGACTTGC | PY03177 418-398 PY03177 544-563 | 60°C annealing/extension; 35 cycles |
| PY05826f PY05826r | AACCGCACAATTAGAACTAG  CTTATGATTGTAGCATAACA | PY05826 66-85 PY05826 215-196 | 60°C annealing/extension; 35 cycles |
| PY01966f PY01966r | ATACGAGGCTTATTGCCCTA TCGGCGAACAAATAATTAAA | PY01966 93-112 PY01966 203-184 | 60°C annealing/extension; 35 cycles |
| PY04021f PY04021r | TGGAAACGACACAACAAATA AGAAGGGGTGGAATATCATC | PY04021 558-577 PY04021 755-736 | 60°C annealing/extension; 35 cycles |
| PY02298 f  PY02298 r | TGCGCAAAATGATATAAATG  GCATCAGGTACAGAACTT | PY02298 207-226  PY02298 356-339 | 60°C annealing/extension; 35 cycles |
| PY03045 f  PY03045 r | AAATGCTGGAGAATTGTTTG  ACGACTACATTCATGGCTATT | PY03045 519-538  PY03045 666-646 | 60°C annealing/extension; 35 cycles |
| Conserved *yir* f1  Conserved *yir* r1 | ATATGGTTAAGTTATATGTTAAACC CCAAATAACGAATACTTATAAGAAATTC | >200 dbase hits with < 2 mismatches | 2/4 mM MgCl_2_  50°C annealing,  45 cycles |
| Conserved *yir* f2  Conserved *yir* r1 | CGATAAAATTAATGCTGGATGTTTA  CCAAATAACGAATACTTATAAGAAATTC | >200 dbase hits with < 2 mismatches | 4 mM MgCl_2_  53°C annealing,  45 cycles |
| Conserved *yir* f2  Conserved *yir* r2 | CGATAAAATTAATGCTGGATGTTTA  GTTTTTGAAATCGTTTCCG | >200 dbase hits with < 2 mismatches | 4 mM MgCl_2_  55°C annealing,  40 cycles |
